# Supplementary material for: Targeted volume imaging reveals early vascular interactions of Lyme disease pathogen in skin
Source: Nat Commun. 2025 Oct 22;16:9330. doi: 10.1038/s41467-025-64326-w (PMC12546650; doi:10.1038/s41467-025-64326-w)
Supplement: Supplementary file 1 — Supplementary Information [file 41467_2025_64326_MOESM1_ESM.pdf]

Targeted volume imaging reveals early vascular interactions of Lyme disease pathogen in skin

Martin Strnad[1,2], Jiří Týč[1], František Kitzberger[1,2], Jana Kopecká[1], Ryan O. M. Rego[1,2] and Marie Vancová[1,2*]

[1] Institute of Parasitology, Biology Centre, Czech Academy of Sciences, České Budějovice, Czech Republic

[2] Faculty of Science, University of South Bohemia, České Budějovice, Czech Republic

* correspondence: vancova@paru.cas.cz

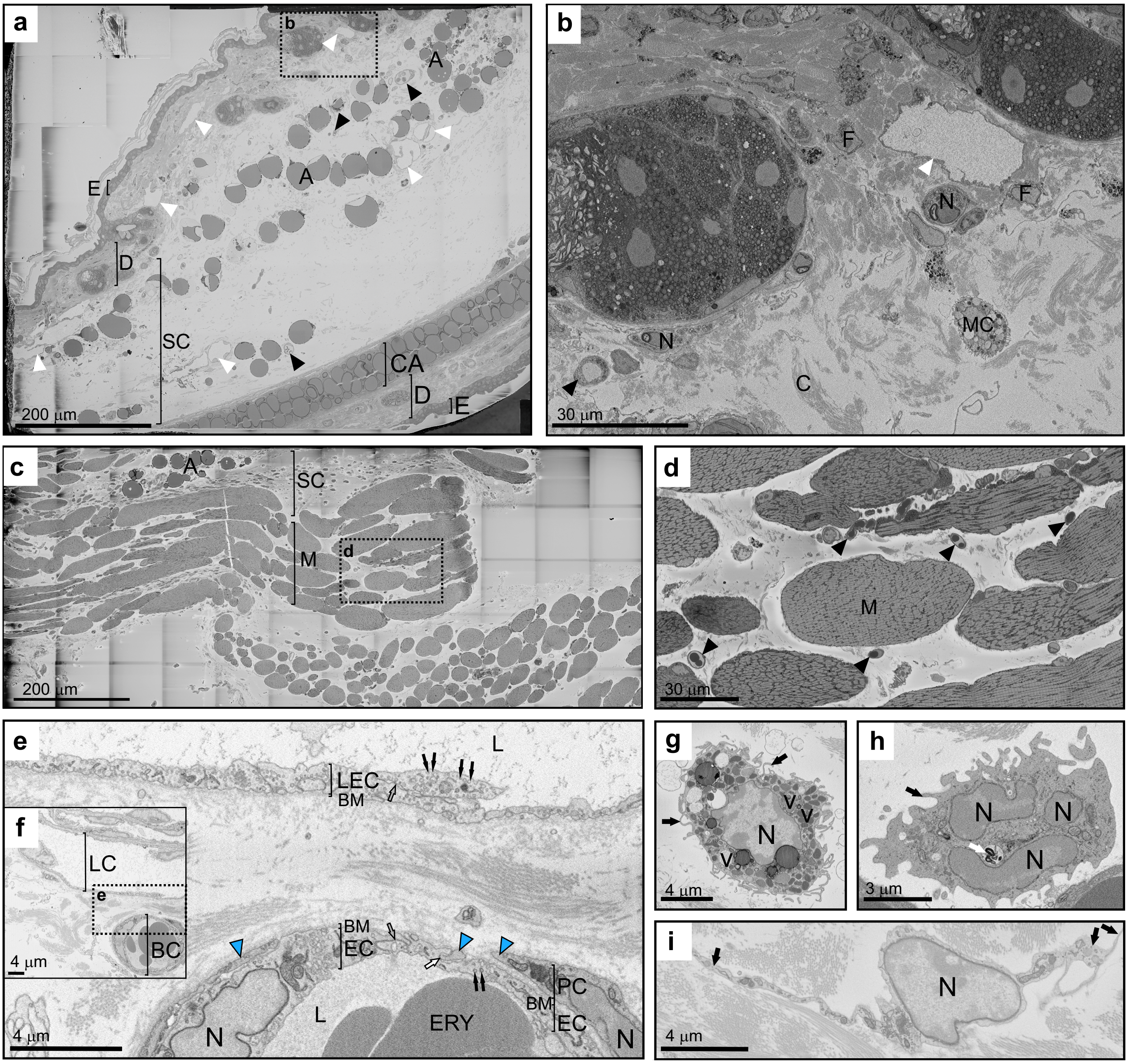

Supplementary Fig. 1. **Representative images from SBF-SEM datasets of mouse tissues infected with *B. burgdorferi*. a, b**, **e – i** Ear. **c, d** Dorsal skin. **a – f** Labeled structures include adipocytes (A), lymphatic capillary (white arrowheads), blood capillary (black arrowheads), cartilage (CA), subcutaneous layer (SC), dermis (D), epidermis (E), nerve (N), mast cell (MC), fibroblast (F), collagen fibers (C), muscle (M). **e, f** Representative images of a lymphatic capillary (LC) and a blood capillary (BC). The BC typically contains erythrocytes (ERY) and other blood cells within its lumen (L). It is lined by endothelial cells (EC) that form electron-dense tight junctions (gray arrow) between neighbouring cells and contain numerous endocytic vesicles (black arrows) in the cytoplasm. The apical surface of ECs is covered by a basement membrane (BM), which is shared with a pericyte (PC). The PC extends long, thin processes (blue arrowheads) that wrap around the BM and may invaginate into the EC (white arrow). The LC is lined by flat lymphatic endothelial cells (LEC) that form outward protrusions (see gray arrows in Fig. 5a). The LC lumen typically appears empty or may contain non-erythrocytic cells. The BM is barely discernible. **g** Representative image of a mast cell with a monolobed nucleus (N), filopodia (black arrows), and cytoplasm densely packed with vesicles (V) of variable electron density and diameter. **h** Representative image of a neutrophil with a multilobed nucleus (N), phagolysosomes (white arrow), filopodia (black arrow), and finely granular, electron-dense cytoplasm; lamellipodia are not visible in this section. **i** Fibroblast with an oval nucleus (N) and, in this section, two long, thin cytoplasmic processes (black arrows), which may be branched or elaborately extended.

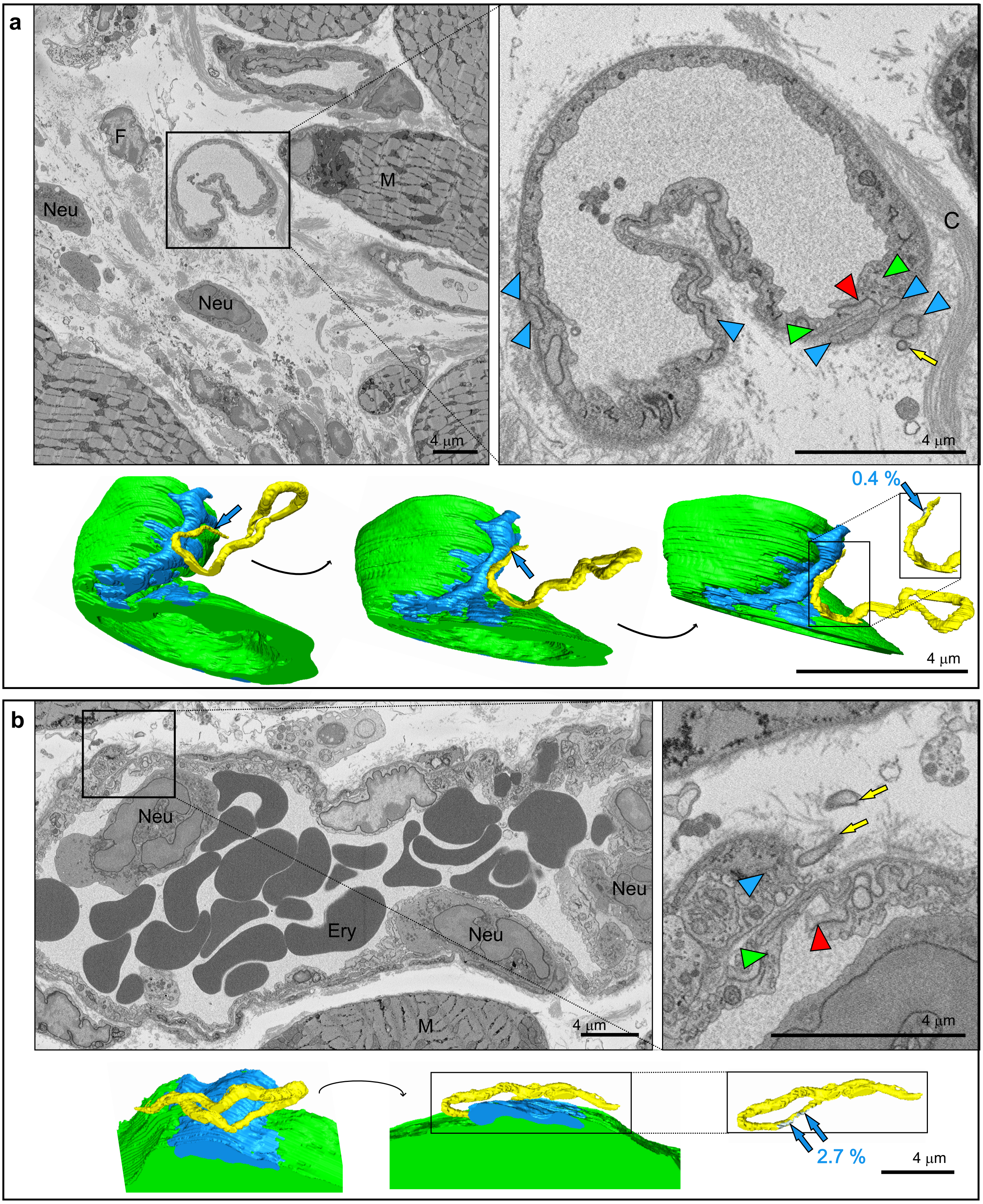

Supplementary Fig. 2. ***B. burgdorferi*** **(*Bb*) in contact with the pericyte projections in the dorsal skin.** Representative slices from SBF-SEM datasets and corresponding 3D models shown from different viewing angles. The percentages represent the *Bb* surface area interacting with the pericyte (blue arrows). **a** Blood capillary. **b** Venule. Structures are marked as follows: *Bb* (yellow, yellow arrows), pericytes (blue, blue arrowheads), endothelial cells (green, green arrowheads), tight junction (red arrowheads), neutrophiles (Neu), erythrocytes (Ery), fibroblast (F), collagen (c), muscle (M).

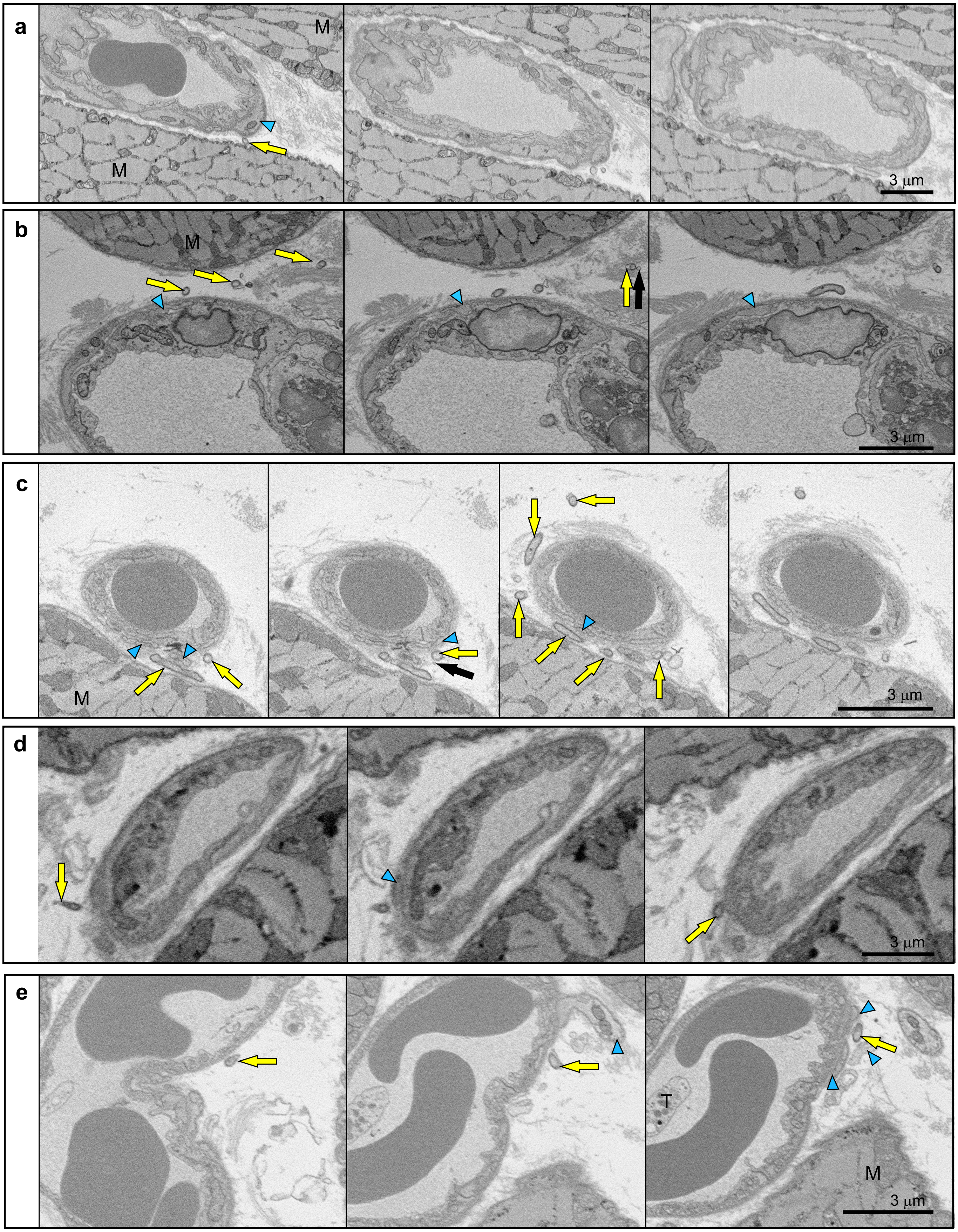

Supplementary Fig. 3. **Intimate contacts of *B. burgdorferi (Bb)* with the blood capillary surface in dorsal skin. a-e** Representative slices from different depths of SBF-SEM datasets. *Bb* is located outside of interstitial collagen bundles in **a**, **e**, but partially inside in **b**, **d**. In **b**, **c**, *Bb* produces outer membrane vesicles (black arrow). Structures are marked as follows: *Bb* (yellow arrows), pericytes (blue arrowheads), muscle (M), thrombocyte (T).

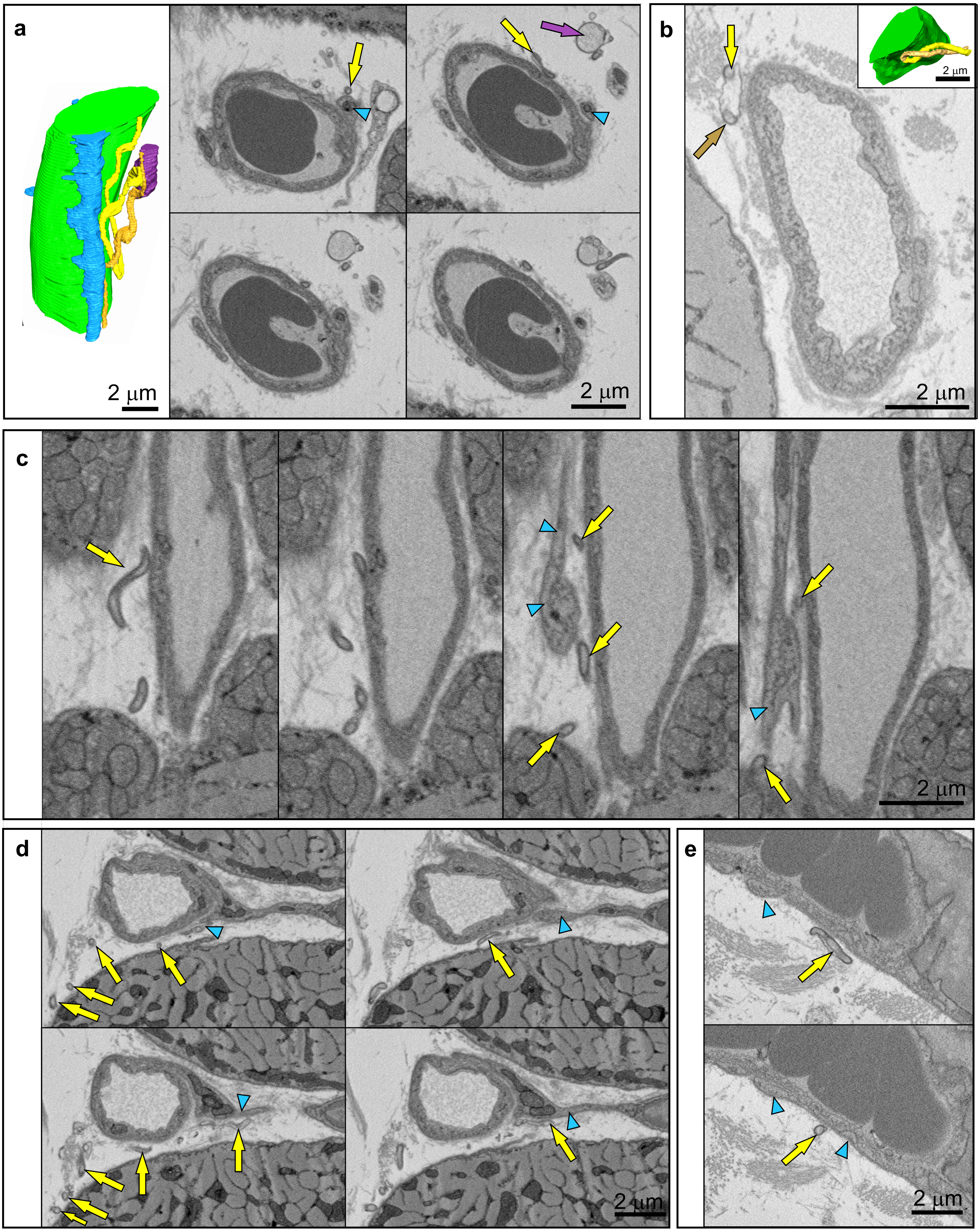

Supplementary Fig. 4. ***Borrelia burgdorferi* (*Bb*) in contact with the blood capillary surface in dorsal skin. a - e** Representative slices from different depths of SBF-SEM datasets. Structures are marked as follows: one *Bb* (yellow arrow), the second *Bb* (orange arrow), pericytes (blue in the 3D model, blue arrowheads), surface of blood capillary (green in the 3D model), atypical structure of *Bb* (violet arrow).

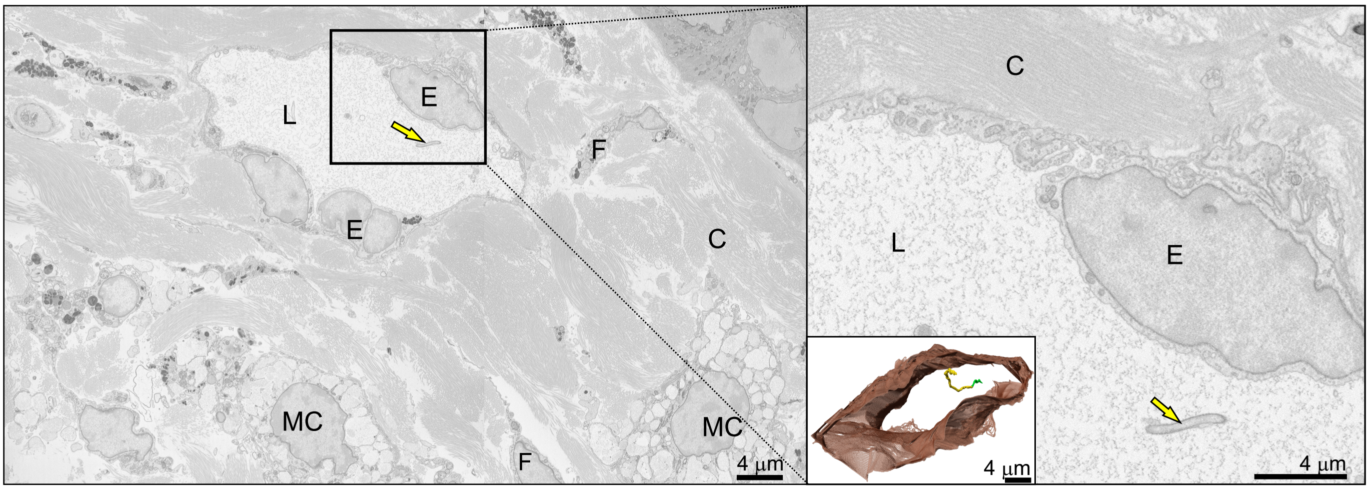

Supplementary Fig. 5. **Visualization of *Borrelia burgdorferi* (*Bb*) in the lumen of the lymphatic capillary.** Overview and detailed slices from the SBF-SEM dataset and the corresponding 3D model show two *Bb* cells (shown in yellow and green). Endothelial cell (E, brown in the 3D model), fibroblast (F), collagen (C), lumen of the lymphatic capillary (L), mast cell (MC), *Bb* (yellow arrow). For further details, see supplementary movie 13.
