# Supplementary material for: Targeted volume imaging reveals early vascular interactions of Lyme disease pathogen in skin
Source: Nat Commun. 2025 Oct 22;16:9330. doi: 10.1038/s41467-025-64326-w (PMC12546650; doi:10.1038/s41467-025-64326-w)
Supplement: Supplementary file 2 — Description of Additional Supplementary Files [file 41467_2025_64326_MOESM2_ESM.pdf]

Title: Supplementary Movie 1

Description: SBF-SEM dataset showing *B. burgdorferi* interactions with a pericyte projection. For further details, see Fig. 2a.

Title: Supplementary Movie 2

Description: SBF-SEM dataset shows *B. burgdorferi* interactions with a pericyte projection. For further details, see Fig. 2b.

Title: Supplementary Movie 3

Description: SBF-SEM dataset demonstrates *B. burgdorferi* interaction with a blood capillary. For further details, see Fig. 4.

Title: Supplementary Movie 4

Description: 3D model based on SBF-SEM data from Supplementary Movie 3. For further details, see Fig. 4. Gray areas represent missing data.

Supplementary Movie 5

Description: SBF-SEM dataset showing *B. burgdorferi* attempting to enter a lymphatic vessel via the transcellular route. For further details, see Fig. 5a.

Title: Supplementary Movie 6

Description: 3D model based on SBF-SEM data from Supplementary Movie 5. For further details, see Fig. 5a.

Title: Supplementary Movie 7

Description: SBF-SEM dataset showing *B. burgdorferi* attempting to enter a lymphatic vessel via the transcellular route. For further details, see Fig. 5b.

Title: Supplementary Movie 8

Description: 3D model based on SBF-SEM data from Supplementary Movie 7. For further details, see Fig. 5b.

Title: Supplementary Movie 9

Description: SBF-SEM dataset showing *B. burgdorferi* attempting to enter a lymphatic vessel via a transcellular route.

Title: Supplementary Movie 10

Description: 3D model based on SBF-SEM data from Supplementary Movie 9.

Title: Supplementary Movie 11

Description: SBF-SEM dataset showing *B. burgdorferi* attempting to enter a lymphatic vessel via a paracellular route. For further details, see Fig. 5c.

Title: Supplementary Movie 12

Description: 3D model based on SBF-SEM data from Supplementary Movie 11. For further details, see Fig. 5c.

Title: Supplementary Movie 13

Description: SBF-SEM dataset showing presence *B. burgdorferi* within the lumen of a lymphatic capillary. For further details, see Supplementary Fig. 5.

Title: Supplementary Movie 14

Description: SBF-SEM dataset showing the presence of *B. burgdorferi* within densely packed collagen fibres. For further details, see Fig. 6.

Title: Supplementary Movie 15

Description: SBF-SEM dataset showing a microvessel with immune cells exiting the bloodstream (arrows). For further details, see Fig. 7b.

Title: Supplementary Movie 16

Description: Coloured SBF-SEM dataset showing immune cell infiltration and neutrophils engulfing *B. burgdorferi*. For further details, see Fig. 7d.

Title: Supplementary Movie 17

Description: 3D model showing a neutrophil actively phagocytosing three spirochetes *B. burgdorferi*. For further details, see Fig. 7d-g.
